# Application of Participatory Design to Co‐Design a Thoracic Surgery Prehabilitation Program

**DOI:** 10.1111/jgs.70141

**Published:** 2025-09-30

**Authors:** Amanda A. Geppert, Sara Kim, Donna Tong, Jane L. Holl, Maria Lucia L. Madariaga

**Affiliations:** ^1^ University of Chicago Medicine Chicago Illinois USA

**Keywords:** design, frailty, prehabilitation, surgery

## 1 Introduction

There is strong evidence that prehabilitation (prehab) before surgery in frail, older adult patients improves perioperative outcomes, yet only 10%–30% of eligible patients adhere to prehab programs [1, 2, 3]. We hypothesize that the lack of “end” user (e.g., patient, caregiver) engagement in the design of prehab programs contributes to poor adherence and describe the first application of user‐centered, participatory, co‐design [4, 5] principles to a prehab program for frail, older adult Thoracic Surgery patients.

## 2 Methods

Patients/caregivers in the thoracic surgery clinic waiting room were invited to participate in a brief (5–10 min) interview to gather feedback on individual components of low‐fidelity, early prototypes (e.g., hand‐drawn pictures) of a prehab program. Three phases of interviews, with new participants at each phase, were conducted with continuous data analysis to inform the next iteration of the prototype (Table 1). For Phase 1, participants were shown an initial prototype of an Apple Watch (with daily exercise reminders and recording physical activity) and multiple formats (e.g., handout, cards, video) of physical activities (e.g., tai chi, walking, Zumba) for patients to perform. For Phase 2, the prototype consisted of the Apple Watch, a prescription for prehab, and a 13‐page, single‐sided, *flipbook* that was branded with the hospital name and included evidence of the benefits of the exercises from professional societies; a page for each exercise with step‐by‐step directions; a daily tracking log; and a brief guide on the use of the Apple Watch. For Phase 3, a near final prototype (Apple Watch, prescription, and *flipbook* with additional refinements to the exercise directions and a “Safety Tips” page) was reviewed by participants and the University of Chicago Health Literacy and Office of Diversity, Equity and Inclusion team. Participants were also asked about their receptivity to wearing the Apple Watch and receiving a daily exercise reminder. Finally, content and layout refinements were made by the study team in alignment with design criteria prioritized by patients and caregivers. The study was approved by the University of Chicago IRB22‐1679.

**TABLE 1:** Prototype testing.

	Phase 1 (9 participants)	Phase 2 (3 participants)	Phase 3 (11 participants)	Final prototype
Description of prototype	Exercise content (e.g., chair exercises, tai chi, walking Zumba) sourced from existing resources: 5‐min core exercise cards for seniors NIA Workout to Go E‐pub SilverSneakers YouTube videos Prototype formats included Apple Watch plus a handout, booklet, deck of cards and/or YouTube video channel.	Physical flipbook for a “Daily prescription” of 3 exercises (marching in place, wall push‐ups, chair squats), and 2 stretches (upper back and calf stretches) for a specific number of weeks, plus Apple Watch Included evidence‐based information from ACS and ASA.	Addition of “safety tips” page and refinement of exercises (names, directions, photography) and tracking logs for each exercise. For step‐by‐step exercise directions, offered participants choice between photography of real people or AI‐generated (ChatGPT) image.	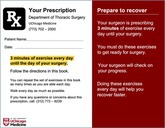 A 15‐page flipbook on stiff heavy‐weight paper and plastic spiral binding to support tenting on a table. Flipbook includes: Breakdown of daily prescription (p. 2) Apple Watch reminders to exercise and track daily steps (p. 3) Safety tips (p. 4) Step‐by‐step instructions for stretches (p. 5–6) Step‐by‐step instructions for exercises (p. 7–9) Log to track daily count for each exercise over 60 days (p. 10–13) Apple Watch instructions (p. 14) Recommendation to walk with a buddy or family member (p. 15)
Expert feedback	A senior researcher in physical therapy suggested: Identifying 3–5 specific exercises. Make exercises of short duration. Order exercises from most to least enjoyable.	A senior researcher in physical therapy suggested: Refined directions. Adding “safety tips” page.	Health literacy team suggested: Using larger font, bolder formatting, larger page dimensions Simplify text to improve readability and understandability.	
Participant feedback to exercise content and instructions	Found activity choices overwhelming. Suggested narrowing choices. Desired evidence‐based activities. Desired activities categorized by difficulty	Liked “Daily prescription” for exercise cover page. Liked simple directions, easy enough to complete at home. Desired info. about amount of physical activity and equipment needed.	Preferred photography of real people to AI‐generated images. Chair‐sit‐to‐stand determined most difficult exercise. Suggested adding walking as social health activity.	
Participant feedback to overall design	Preferred large‐format paper tool. Suggested use of institutional branding to convey quality. Include paper version of exercise log to “check off” when completed.	Liked physical tool.	Asked for consolidated exercise log instead of separate logs for each exercise. Desired better flipbook durability.	
Participant feedback to reminders	Receptive to receipt of daily Apple Watch reminders to exercise and to log exercises, with some requesting customization option. Receptive to weekly “check‐in” by Thoracic Surgery staff by phone or text.			

## 3 Results

**FIGURE 1:**
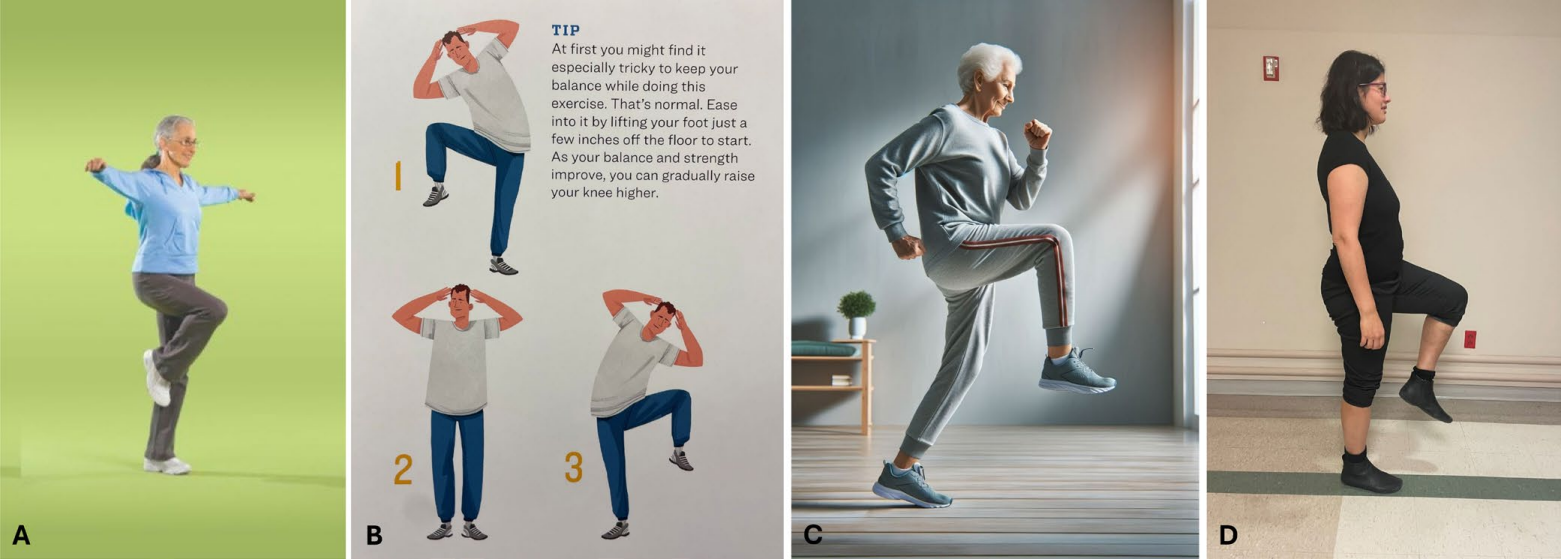
Examples of iterations for exercise instruction images: (A) NIA Go4Life (B) 5‐min core exercise cards for seniors by Cindy Brehse, sourcebooks (C) Chat GPT AI image (D) final image.

A total of 19 patients and 4 caregivers (*n* = 23) participated in the interviews (participation rate 96%, 23/24). Feedback from Phase 1 participants (*n* = 9) indicated a desire for (1) a “booklet” that is institutionally “branded” to convey quality and includes a limited number of exercises with evidence of patient benefit and is ordered by difficulty and (2) a “prescription” for prehab. Phase 2 participants (*n* = 3) endorsed the *flipbook* (booklet), found the exercise directions simple enough to complete at home, and requested more information about needed equipment. Phase 3 participants (*n* = 11) offered nuanced feedback about the content, order, and directions of the exercises (e.g., how to address poor balance or low strength; how to increase intensity), suggested adding walking as a physical activity, recommended modifying the log with a column for each exercise, expressed a preference for the photographs of directions to be of actual people rather than ChatGPT‐generated images (Figure 1), and recommended using heavier paper to assure durability. All participants were receptive to wearing the Apple Watch; some requested an option for “customized” reminder times; and some wanted a weekly “check‐in” by Thoracic Surgery staff by phone or text. The final review led to the use of a larger font, bolder formatting, larger page dimensions, and simplified text to improve readability and understandability.

## 4 Discussion

The study reveals that patients/caregivers can offer substantial design recommendations for a prehab program. The high participation rate suggests that participatory co‐design is highly feasible, even with older frail adults. Patient/caregiver participants can offer unique, previously unrecognized modifications to address their needs, preferences, and values.

The study is limited by gathering feedback from a limited number of participants at a single institution despite its highly diverse patient population and by a focus on exercise only, although prehab programs can include additional (e.g., nutrition, sleep) components. Evaluation of the prehab program on adherence and outcomes is underway.

## Author Contributions

Study concept and design: M.L.L.M. and J.L.H. Acquisition of subjects and/or data: S.K. and A.A.G. Analysis and interpretation of data: M.L.L.M., J.L.H., S.K., A.A.G., and D.T. Preparation of manuscript: M.L.L.M., J.L.H., S.K., A.A.G., and D.T.

## Disclosure

The content is solely the responsibility of the authors and does not necessarily represent the official views of the National Institutes of Health.

## Conflicts of Interest

The authors declare no conflicts of interest.

## References

[1] F. Carli , G. Bousquet‐Dion , R. Awasthi , et al., “Effect of Multimodal Prehabilitation vs Postoperative Rehabilitation on 30‐Day Postoperative Complications for Frail Patients Undergoing Resection of Colorectal Cancer: A Randomized Clinical Trial,” JAMA Surgery 155, no. 3 (2020): 233–242. 31968063 10.1001/jamasurg.2019.5474 PMC6990653

[2] D. I. McIsaac , M. Gill , L. Boland , et al., “Prehabilitation in Adult Patients Undergoing Surgery: An Umbrella Review of Systematic Reviews,” British Journal of Anaesthesia 128, no. 2 (2022): 244–257. 34922735 10.1016/j.bja.2021.11.014

[3] D. I. McIsaac , E. Hladkowicz , G. L. Bryson , et al., “Home‐Based Prehabilitation With Exercise to Improve Postoperative Recovery for Older Adults With Frailty Having Cancer Surgery: The PREHAB Randomised Clinical Trial,” British Journal of Anaesthesia 129, no. 1 (2022): 41–48. 35589429 10.1016/j.bja.2022.04.006

[4] E. Chen , G. Neta , and M. C. Roberts , “Complementary Approaches to Problem Solving in Healthcare and Public Health: Implementation Science and Human‐Centered Design,” Translational Behavioral Medicine 11, no. 5 (2021): 1115–1121. 32986098 10.1093/tbm/ibaa079 PMC8158168

[5] R. C. Smith , D. Loi , and H. Winschiers‐Theophilus , Routledge International Handbook of Contemporary Participatory Design (Routledge, 2025).

